# Editorial: Nutrition and chemistry of cereal macromolecules in cereal-based products

**DOI:** 10.3389/fnut.2022.1097060

**Published:** 2022-11-30

**Authors:** Hua-Min Liu, Sen Ma, Hong-Shun Yang, Kunlun Liu

**Affiliations:** ^1^College of Food Science and Engineering, Henan University of Technology, Zhengzhou, China; ^2^Department of Food Science and Technology, National University of Singapore, Singapore, Singapore; ^3^School of Food and Strategic Reserves, Henan University of Technology, Zhengzhou, China

**Keywords:** cereal, macromolecules, chemistry, nutrition, cereal-based products

Cereals belong to the family of Gramineae, which produce dry, one-seeded fruits called grains that consist of seed and pericarp. The seed itself consists of the seed coat, the nucellar epidermis, the endosperm, and the embryo. Recently, cereals and their ingredients are accepted as nutraceuticals and functional food due to providing dietary fiber, energy, proteins, vitamins, minerals, and phytochemicals required for human health. Nutrition and chemistry of cereals and cereal-based food are the pillars of wellbeing and health in society because humans rely on cereals not only to supply energy but also for essential nutrients that maintain the body and keep the immune system in a good state of recovery. Adequate nutrition, therefore, correlates with lower mortality and morbidity from both non-infectious and infectious diseases and is particularly important in pregnant women and children where the lack of essential nutrients can result in irreversible mental and physical damage during development.

Nowadays, the nutrition and chemistry of cereals and its processed products are a major priority to human health. The main compositions in cereal and cereal-based products are carbohydrate (starch and structural compositions), lipid, and protein, which belong to macromolecules and provide basic nutrition for humans and animals. Therefore, given the significance of the project, the journal has been seeking original research papers and review articles to organize a Research Topic focused on *Nutrition and chemistry of cereal macromolecules in cereal-based products*. After 6 months of preparation, we believe that this Research Topic is ready to be shared with researchers around the world. We have received many research papers and review articles since the opening of the submission system. The interesting ideas, unique insights and positive feedback from all researchers were very impressive in preparation for the Research Topic. Excitingly, finally, there are nine papers covering almost all features of *Nutrition and chemistry of cereal macromolecules in cereal-based products* and they provide an in-depth understanding of the techniques and methodologies used in the journal.

Firstly, scientists investigated the functional and nutritional changes of macromolecules in cereal-based foods after fermentation and enzyme treatment. For example, the paper from Liu X. et al. investigated the effects of fermented and germinated foxtail millet whole grain (FG-FM) on kidney lesions in a diabetic mouse model (Db/Db mice). The paper found that the FG-FM consumption significantly alleviated the kidney tissue damage in the diabetic mouse model. They also concluded that the over activation of signaling pathways related to inflammation and immunity in the diabetic mouse model was significantly inhibited with the FG-FM intake. This investigation confirmed foxtail millet as a potential source of functional food for the non-pharmacological intervention of DKD. The second paper (Fan et al.) evaluated the effect of fermented wheat bran dietary fiber (FWBDF) on the rheological properties of the dough and the quality of noodles and to compare it with the effect of the unfermented WBDF (UWBDF). It revealed that fermentation could reduce the destructive effects of WBDF on the quality of noodles, providing a new perspective on balancing dietary fiber-rich and high-quality foods. The third paper (Qin et al.) investigated the transglutaminase treatments on the structure of pea protein isolate under conditions relevant to high-moisture extrusion processing by using a closed cavity rheometer. The findings can help to better understand the relationships of material-structure during the extrusion process, and also provide guidance for further optimization of the quality of meat substitutes. Wang et al. discussed the structural properties and aggregation behavior of gluten containing wheat bran dietary fiber under the conditions of synergistic fermentation of *Lactobacillus plantarum* and *Saccharomyces cerevisiae*, which provides new data for the improved production of sourdough whole grain and/or high fiber flour products. Liang et al. investigated the physicochemical and functional properties of soybean protein concentrate by using Alcalase protease and high-pressure homogenization for the combined modification. The results indicated the modification technology could improve the functionality and application range of soybean protein concentrate, which could also provide a theoretical basis for its high-value utilization in food industry.

Secondly, four articles present the functional and structural properties of starch or starch-based foods during processing. Guo et al. determined the effect of three kinds of natural antioxidants on the structural and physicochemical properties of sweet potato starch in noodles. They found that the broken rates, iodine blue values, hardness, and chewiness of the noodles were increased with the addition of the tested natural antioxidants. Additionally, the adding natural antioxidants could improve the sensory quality and antioxidant function of starch noodles. Liu G. et al. analyzed the oral processing properties and the starch fine structure of japonica rice. Additionally, the relationship between starch fine structure and oral processing of cooked japonica rice was further investigated. Ji et al. studied the pasting, retrogradation, and structural properties of three different crystalline starches compounded with natural inulin. The potential mechanism of interaction between inulin and starch was also investigated. The results could develop the theoretical system of inulin with starch compound system and could provide a solid theoretical basis of further applications. Yan et al. modified wheat flour by annealing using plasma-activated water. This method has a potential for further application in wheat flour modification as a green technology.

In conclusion, the collections in this Research Topic covers the effects of cereal macromolecules including starch, polysaccharide, and protein on the functional and nutritional properties of cereal-based foods during processing. We hope that this Research Topic will further promote the interests in *Nutrition and chemistry of cereal macromolecules in cereal-based products*.

## Author contributions

H-ML and KL prepared, checked, and revised the manuscript and approved the submitted version. All authors contributed to manuscript revision, read, and approved the submitted version.

## Conflict of interest

The authors declare that the research was conducted in the absence of any commercial or financial relationships that could be construed as a potential conflict of interest.

## Publisher's note

All claims expressed in this article are solely those of the authors and do not necessarily represent those of their affiliated organizations, or those of the publisher, the editors and the reviewers. Any product that may be evaluated in this article, or claim that may be made by its manufacturer, is not guaranteed or endorsed by the publisher.

